# Aluminum Foam Sandwich: Pore Evolution Mechanism Investigation and Engineering Preparing Optimization

**DOI:** 10.3390/ma16196479

**Published:** 2023-09-29

**Authors:** Xi Sun, Zhiqian Jian, Xixi Su, Peng Huang, Qiang Gao, Zhanhao Feng, Guoyin Zu

**Affiliations:** 1 School of Metallurgy, Northeastern University, Shenyang 110819, China; sunxi0524@163.com; 2 School of Materials Science and Engineering, Northeastern University, Shenyang 110819, China; jianzq18437932722@163.com (Z.J.); suxi9573@163.com (X.S.); neuhuangpeng@163.com (P.H.); 2110253@stu.neu.edu.cn (Q.G.); zhfengsincerity@gmail.com (Z.F.)

**Keywords:** aluminum foam sandwich, pore evolution mechanism, engineering preparation, cavity design, plate shape control

## Abstract

This paper employs an innovative investigation approach to study pore evolution in Al-Si-Mg-Cu alloy within aluminum foam sandwiches (AFS) by integrating data from heating–expansion ratio curves, in situ observation of synchronous radiation, and microscopic analysis of the matrix’s microstructure at different stages. Additionally, the cavity design and plate type control for large-scale AFS production are explored. Findings categorize the precursor heating into three stages: rapid heating, solid–liquid transition, and stable foaming. During solid–liquid transition, the expansion rate experiences a sudden drop, associated with pore nucleation and edge cracking of precursors. Pores nucleate as elongated crack-like structures along the rolling direction, guided by the Mg-enriched regions. In stable foaming, these pores evolve, become spherical, and the matrix rapidly expands. Using square tubes for sealing on the preform cavity sides creates a dense edge zone during rolling, halting crack propagation into the powder core. Adopting edge sealing during foaming mitigates boundary effects, thereby improving AFS panel flatness.

## 1. Introduction

Aluminum foam sandwich panels (AFS) belong to an innovative category of porous composite materials renowned for their exceptional physical attributes [1]. These properties encompass a lightweight design, high specific stiffness and strength [2], proficient energy absorption [3,4], effective sound insulation capabilities [5,6], and adept electromagnetic shielding [7]. The applications span aerospace and transportation sectors, where lightweight designs are crucial [8], urban construction for creating noise-reducing structures, and the military industry, where AFS serves in energy-absorbing components for armored vehicle chassis [9].

Powder metallurgy foaming stands as one of the primary preparation methods, enabling the metallurgical bonding of metal panels with porous core layers, thus endowing them with exceptional performance. However, when compared to aluminum foam produced through the melt foaming method, the powder metallurgy foaming process requires further optimization to maintain a stable foaming environment, regulate cell uniformity, and rectify core layer defects. Among them, a crucial issue to address is ensuring the compatibility between the foaming agent’s decomposition temperature and the melting temperature of the core layer powder [10,11]. Researchers have enhanced the decomposition temperature by oxidizing TiH_2_ to reduce gas losses during the foaming process [12,13]. Simultaneously, reducing the melting temperature of the matrix presents an effective approach to addressing temperature mismatches. In the Al-Si series matrix, pore nucleation predominantly occurs at the Al-Si interface [14], where elevated internal pressure during nucleation leads to the loss of hydrogen gas, resulting in an uneven distribution of pores. This pore nonuniformity issue is mitigated when using the Al-Si-Cu series with a lower melting temperature [15,16]. Additionally, researchers have found that employing the Al-Si-Mg series as the matrix results in a significant amount of melting within a narrow temperature range, facilitating the formation of an excellent expansion and foam structure [17]. Moreover, Ding et al. [18,19] noted that the introduction of Sn further reduces the foaming temperature and enhances the foam structure in the Al-Si-Mg-Cu series. In these alloy series, the Al-Si-Mg-Cu series is used in the commercial manufacturing of AFS. [20]. Nevertheless, the cell evolution mechanisms for this specific alloy composition remain unclear.

Furthermore, during the engineering process of producing precursors through pack rolling, disparities in properties between solid metal and core layer powder, coupled with variations in their deformation behavior, can pose various challenges. These challenges encompass diminished powder densities and the occurrence of edge cracking defects, ultimately resulting in compromised foaming properties and increased production costs [21,22]. Moreover, shaping the plate flatness during the foaming process represents a critical technical challenge in the pursuit of successful industrial-grade production of large-sized AFS.

In this paper, the method of combing thermal expansion data, in situ pore evolution observations using synchrotron radiation, and microstructural analyses at various stages is employed to elucidate the foaming mechanism in the production of Al-Si-Mg-Cu alloy metal foams. In addition, the formation mechanism of edge crack defects in precursor was investigated by comparing different cavity side sealing methods. Furthermore, the reasons affecting the flatness of the AFS plate shape are analyzed, and solution strategies are proposed. This work provides vital theoretical guidance for further enhancing the engineering preparation process of AFS.

## 2. Experimental Procedures

**Figure 1:**
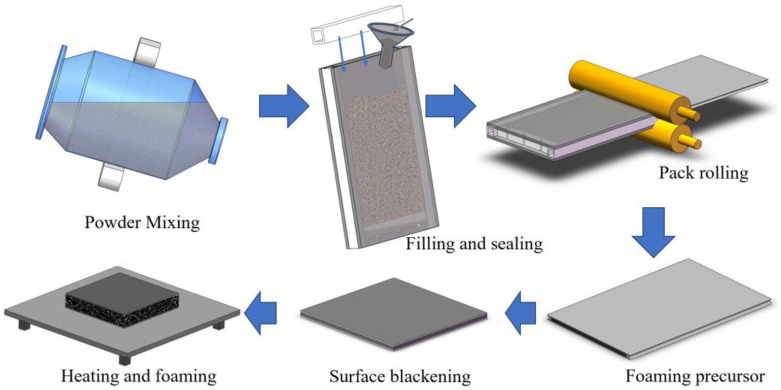
Schematic diagram of preparing AFS.

**Figure 2:**
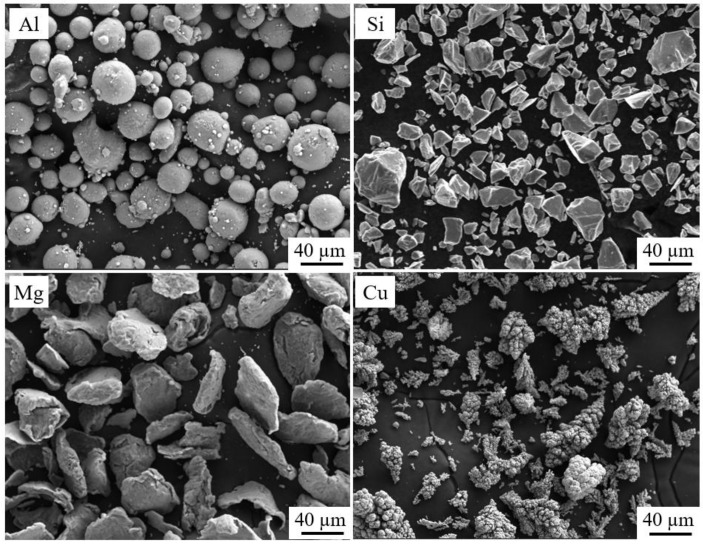
SEM micromorphology of Al, Si, Mg, and Cu powder particles.

The production of AFS involves a pack rolling process, as depicted in Figure 1. This process consists of several sequential steps, starting with powder mixing and sealing, followed by cold rolling, hot rolling, panel treatment, and the foaming process. The foaming matrix is a mixture of pure Al, Si, Mg, and Cu powders with AlSi_6_Mg_4_Cu_4_ composition. The SEM micromorphology of these powders is shown in Figure 2. Additionally, the foaming agent, titanium hydride, is added at 1 wt.%, which has been preoxidized at 470 °C for 3 h. Achieving a homogeneous powder blend is essential, and we accomplish this by mixing these powders in an SYH-600 three-dimensional mixer (Changzhou Yi Neng Drying Co., Ltd., Changzhou, China) for 2 h.

### 2.1. Cavity Optimization Design for Preforms

**Figure 3:**
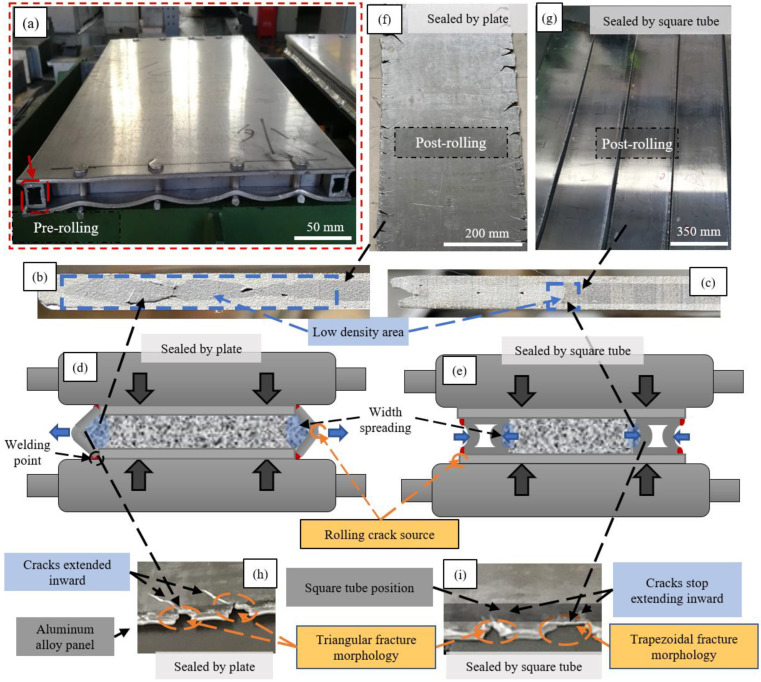
Cavity design and optimization mechanism. (**a**) Pre-rolling preforms sealed by square tubes; (**b**,**c**) Cross-sectional structure of post-rolled preforms sealed by plates/square tubes; (**d**,**e**) Deformation behavior of preforms sealed by plates/square tubes during rolling; (**f**,**g**)Overall appearance of post-rolling preforms sealed by plates/square tubes; (**h**,**i**) Edge crack characteristics in preforms sealed by plates/square tubes.

The successful engineering-grade production of AFS critically depends on the meticulous optimization of cavity design. Figure 3a illustrates the sealed preform, consisting of upper and lower panels crafted from 3003 aluminum alloy, each with dimensions of 4 × 350 × 600 mm^3^. To achieve edge sealing, square tubes of 6063 aluminum alloy measuring 20 × 20 × 3 mm^3^ were utilized. These tubes were welded along the rolling direction between the edge of the upper and lower panels using a flux of ER5356 (red circles in Figure 3a), forming a tubular cavity. The cross-sectional dimensions of this cavity are 20 mm (height) × 300 mm (width), facilitating powder filling. Similar square aluminum tubes were employed as sealing components at both ends of the tubular cavity. After filling the mixed metal powder into the cavity and securing it, the ends of the tubular cavity were riveted to ensure comprehensive sealing. The asbestos cloth was strategically placed at the sealing site, permitting residual gas to escape through rivet gaps during rolling while stopping the powder from escaping. This measure effectively prevents the splattering of matrix powder during the rolling process, thereby reducing powder loss and minimizing environmental contamination.

During the rolling process, the powder within the mold cavity undergoes width spreading at the cavity’s edges. However, as rolling deformation increases, the flowability of the powder diminishes, impeding the effective transfer of the powder from the central to the edge regions. Consequently, this leads to the formation of low-density regions of powder core with poor plasticity along the edges of the mold cavity, as the blue areas marked in Figure 3d,e. Additionally, adhering to the constant volume principle, width spreading induces less deformation at the precursor’s edges along the rolling direction compared to the central region, resulting in tensile stresses at the edges. Consequently, internal fractures occurred within the edge of the precursor’s powder core. The outer panel then embedded itself within these fractures, creating a wavy morphology at the interface between the panel and the powder core. 

The aforementioned phenomenon is clearly demonstrated by the cross-section of the sample in Figure 3b, where aluminum plates were used for the edges. In contrast, Figure 3c presents the outcomes of rolling a preform with square tubes as sealing materials. This configuration significantly reduces the extent of wavy composite interfaces compared to the former. Thus, the utilization of square tubes for sealing on both sides of the preform effectively reduces the formation of low-density areas with poor edge foaming ability, thereby improving powder utilization. 

This reduction is attributed to the deformation characteristics of square tubes, which effectively decrease the extent of width spreading of powder during the rolling process, as depicted in Figure 3e. In contrast to the deformation pattern observed when plates were employed as side sealing materials (as depicted in Figure 3d), the cross-sectional area with bulging features at the cavity edges is smaller. Thus, powder core utilization increases as the low-density area decreases.

The utilization of square tubes as sealing materials on both sides of the preform results in a noteworthy reduction in edge cracking during the rolling process, ensuring a seamless rolling experience. This effect is vividly portrayed in Figure 3g, which presents rolled plate preforms with an elongation rate exceeding 300%. Notably, the side edges remain intact post-rolling, devoid of any prominent instances of edge cracking. In contrast, Figure 3f displays side edges sealed using welded plates, leading to evident edge cracking within the rolled plate preform and suboptimal cavity sealing. Figure 3h offers a visual representation of the edge cracking phenomenon observed in the plate preform after rolling with plates as sealing materials. As rolling elongation increases, crack initiation points appear at locations highlighted by yellow circles in Figure 3h, subsequently propagating into the core powder. In severe cases, this progression culminates in a complete fracture of the plate preform during rolling. Figure 3i showcases edge cracking in the plate preform post-rolling with square tubes as sealing materials. As rolling elongation increases, crack initiation points manifest at the yellow-circled regions. However, the densely packed square tubes effectively hinder the cracks from propagating into the powder core. Evidently, the advancement of the crack is successfully halted at the periphery of the gray-shaded region, precisely where the square tubes are tightly consolidated. Some cracks even exhibit a trapezoidal shape, indicating their inability to further penetrate the powder. 

### 2.2. Rolling Process

The compaction process of the powder entails two distinct stages: an initial cold rolling phase followed by subsequent hot rolling. After sealing the powder, a significant amount of air became trapped among the particles, contributing to favorable powder flowability with a powder relative density of approximately 60% (1.7 g/cm^3^). Meanwhile, the distribution of powder within the mold cavity during the filling process was influenced by gravity, leading to inherent gradients. In response, a meticulously planned, multipass, low-strain-rate cold rolling process was carried out at room temperature. This deliberate approach achieved uniform powder distribution while avoiding powder splatter, facilitating the expulsion of entrained air, and reducing the possibility of powder oxidation during the subsequent high-temperature rolling stage. Throughout each pass of the cold rolling process, a strain rate was meticulously maintained at or below 2% while the rolling speed remained under 5 m/min. The cold rolling procedure concluded once a significant elongation was achieved along the rolling direction. Its total maximum strain is predominantly dictated by the density at which the powder is packed.

**Figure 4:**
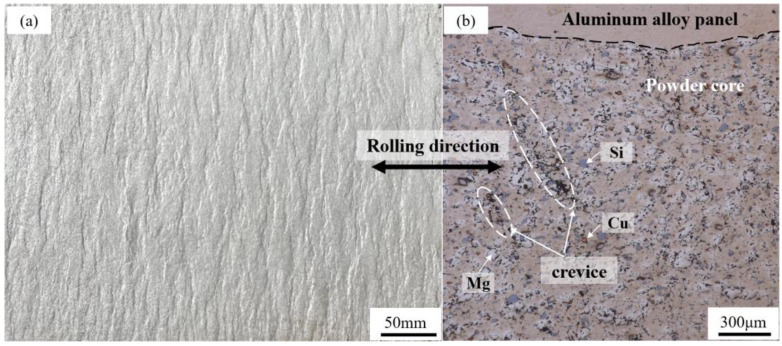
AFS produced by cold rolling process: (**a**) corrugated bonding interface and (**b**) microstructure.

The relative density (*d*) of the powder after cold rolling is calculated using the following formula:

(1) d=MH/ρhV×100%

where *M* is powder filling mass, *V* is mold cavity volume, *H* is initial precursor thickness, *h* is precursor thickness after cold rolling, and *ρ* is average mixed powder particle density. Experimental results indicate that the powder’s relative density (*d*) after cold rolling approximates 85% (2.4 g/cm^3^), implying around 15% compressible space within the core layer. Continued cold rolling, however, can lead to a macroscopic wavy composite interface morphology between the panel and powder core along the rolling direction (Figure 4a), marked by weak bonding and susceptibility to stripping. Microscopically, the powder particles exhibit weak bonding capability, with observed cracks in the powder core, as shown in Figure 4b.

Therefore, when the preform initiates its extension in the rolling direction, an additional hot rolling procedure becomes necessary. This procedure aims to enhance the density of the powder core and establish a strong bond between the outer panel and the powder core. Notably, the oxide film at the aluminum alloy’s surface will impede the high-temperature diffusion process at the panel–foam core interface afterward. Hence, it is essential to ensure adequate elongation during rolling, fostering the rupture of the oxide film at the panel-core junction. This rupture is achieved through a synergistic combination of rolling pressure and friction with the powder surface. Furthermore, increasing the level of rolling deformation is crucial for the powder core, as it elevates the core powder’s density and augments bonding strength among its particles. Therefore, a total reduction exceeding 75% was implemented, corresponding to a total elongation surpassing 300% along the rolling direction. Extensive experience indicates that while maintaining a constant overall deformation, higher deformation in a single pass contributed to a more uniform distribution of foam pores after the precursor’s foaming process. However, it remains crucial to avoid edge cracking during rolling with a cap on deformation per pass set at 40%. The rolling temperature was meticulously controlled between 330 and 420 °C, while the rolling speed was maintained at 10 m/min. Ultimately, foamed precursors with thicknesses of 3.5–6.5 mm were produced.

### 2.3. Foaming Experiments

**Figure 5:**
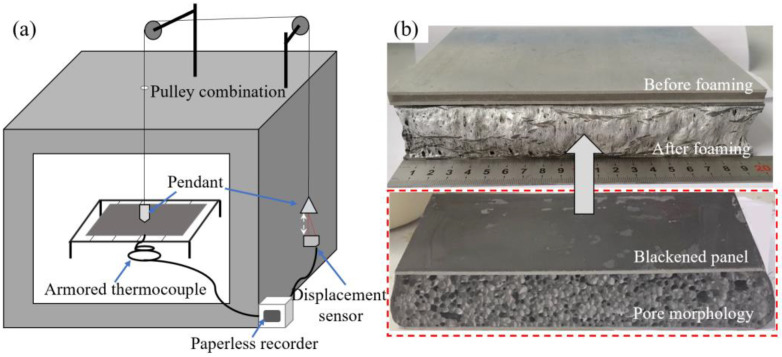
(**a**) Schematic diagram of foaming device; (**b**) precursor before foaming and AFS after foaming.

Foaming experiments were carried out using a custom-designed apparatus capable of real-time monitoring of time, temperature, and expansion ratio during the precursor foaming process, as illustrated in Figure 5a. Its height-measuring device consisted of pulley blocks, asbestos wire, pendants, and a high-precision infrared displacement sensor, which achieved a low measurement error of repetition accuracy within ±35 μm. In Figure 5b, we showcase the precursor both before and after foaming. It is worth noting that the precursor’s panel underwent a blackening treatment to enhance its absorption of infrared radiation energy within the furnace, thus enabling rapid heating. This treatment contributed to the achievement of an excellent pore structure, as demonstrated. The expansion rate is calculated using the following formula:

*Γ* = (*H* − 2*b*)/(*h* − 2*b*) × 100% (2)

where *H* represents the total height after foaming, *b* indicates the panel thickness, and *h* denotes the total height before foaming.

### 2.4. Characterization

To explore the internal macrostructure of the produced aluminum foam, wire electrical discharge machining (EDM) was employed to create cross-sectional cuts in various orientations. These cuts were then painted, polished, and subjected to optical imaging, followed by binary image processing. SolidWorks is utilized to construct a three-dimensional model by splicing the aforementioned binary image, maintaining a 1:1 scale with the actual sample dimensions. The microstructure of AFS at different foaming stages was investigated using a scanning electron microscope (SEM) equipped with an energy dispersive spectrometer (EDS). Furthermore, the dynamic evolution of foam pores during the powder metallurgy foaming process of AFS was observed through the Beijing 4W1A synchrotron radiation imaging station [23]. For this real-time imaging, the sample thickness was limited to 1.5 mm, enabling the synchrotron radiation light source to penetrate the sample and ensure high-quality imaging. 

## 3. Results and Discussion

### 3.1. Pore Structure Evolution Mechanisms

During the foaming process of AFS precursors, unique patterns emerge in the heating rate and expansion ratio as the foaming time progresses. To elucidate these characteristics at specific temperatures, experiments are conducted using a graphite-coated treated precursor measuring 120 × 120 × 6.5 mm^3^ at 600 °C. The resulting time–temperature–expansion ratio and temperature–expansion ratio curves are generated. 

**Figure 6:**
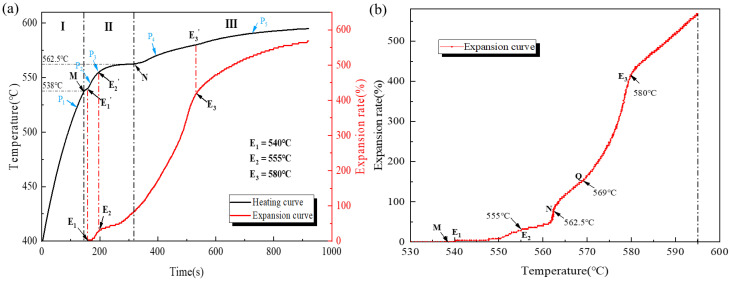
(**a**) The time, temperature, and expansion curve; (**b**) temperature and expansion rate curve.

By analyzing the heating rate curve in Figure 6a, three distinct stages are discerned: (Ⅰ) rapid heating, (Ⅱ) solid–liquid transition, and (Ⅲ) stable foaming. Points M and N are identified as the division markers. At point M, fluctuations in the heating curve commence, marking the initiation of the solid–liquid phase transition. Prior to point M, the heating rate was swift, and the powder core remained solid. Despite the temperature surpassing the initial decomposition point of titanium hydride, no significant expansion occurs in the precursor. As the temperature progresses from M to N, the heating rate gradually declines due to the heat absorption of increasing powder alloying, leading to the matrix transitioning from solid to liquid. Particularly, the heating rate approaches zero when the temperature reaches point N. At this point, the matrix achieves a balance between its heat absorption efficiency from the infrared radiation in the furnace and the energy density required for its solid–liquid transition. We define point N as the plateau temperature. As the temperature reaches above point N, the precursor enters the stable foaming stage. Compared to the former stage, the heating rate exhibits a noticeable increase, and the proportion of the liquid phase in the foam matrix is much higher. Subsequent to this, the heating rate gradually decreases as the temperature difference between the sample surface and furnace temperature lessens. 

Analyzing the expansion rate curve in Figure 6a, matrix expansion initiates at point E_1_. The temperature range between E_1_′ (corresponding temperature of E_1_ point on heating curve) and E_2_′ (corresponding temperature of E_2_ point on heating curve) exhibits the highest expansion rate, followed by a decline after surpassing E_2_, attributed to the release of accumulated gas pressure from early titanium hydride decomposition. From E_2_′ to N, despite the matrix heating rate decline, the expansion rate increases. This can be attributed to the significant increase in the liquid-to-solid ratio of the matrix, leading to a reduction in its deformation resistance, along with the intensified decomposition effect of titanium hydride. Point E_3_ demarcates an inflection, signifying an increasing expansion rate up to E_3_ and a subsequent decrease. 

Figure 6b depicts the expansion rate and real-time temperature relationship. Notably, the expansion rate exhibits a minimum at E_2_ and a maximum at N. The expansion process reveals three increasing intervals, E_1_ to E_2_, E_2_ to N, and Q to E_3_, interspersed with two decreasing intervals, N to Q, and temperatures above E_3_.

**Figure 7:**
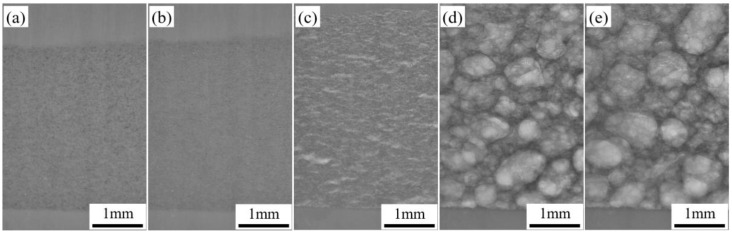
Powder metallurgy foaming process observed in synchrotron radiation field: (**a**) before temperature point M; (**b**) at temperature point E_2_′; (**c**) in the temperature range from E_2_′ to N; (**d**,**e**) above temperature point N.

To investigate the transformation of the pore structure as the precursor underwent heating, dynamic imaging of the AFS powder metallurgy foaming process was conducted using synchrotron radiation, as portrayed in Figure 7. A heating rate similar to the curve shown in Figure 6a was adopted during foaming. In Figure 7a, the state of the foaming precursor before reaching temperature point M within the synchrotron radiation field is depicted. At this stage, the powder core layer exhibits high density and tight bonding with the outer panel. Copper particles, distinguished by their higher atomic numbers, appear as dark entities in the imaging field. No significant changes occur within the powder core during this initial heating phase. As the temperature advances, the copper particles gradually lighten in the synchrotron field. This phenomenon signifies their role as nucleation sites for the formation of pores. Subsequently, the matrix begins to expand, with its expansion rate gradually increasing. Then, a transient decreasing process in the expansion rate is observed in the synchrotron field, corresponding to the trend observed at point E_2_ on the expansion rate curve, as illustrated in Figure 7b.

In the ensuing temperature range from E_2_′ to N, the matrix’s expansion rate experiences another acceleration. During this phase, the matrix possesses a lower liquid-to-solid ratio, imposing limitations on its expansion. This results in the formation of prominent crack-like features within the evolving pore structure, oriented to the rolling direction, as demonstrated in Figure 7c. As the warming process continues, the foaming environment improves due to an increased liquid-to-solid ratio. This improvement leads to enhanced pore sphericity, as shown in Figure 7d. The pore walls undergo creep deformation and rupture due to the ongoing decomposition of titanium hydride with rising temperatures. This prompts pore growth as they merge. These processes collectively contribute to the growth of pore diameter during the stable foaming period, as illustrated in Figure 7d,e.

**Figure 8:**
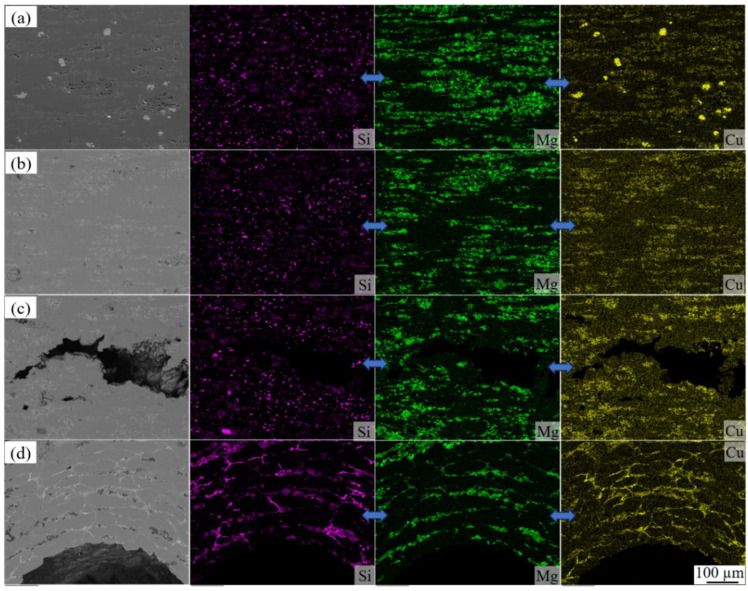
Distribution of Si, Mg, and Cu elements within the matrix corresponding to temperature points (**a**) P_1_, (**b**) P_2_, (**c**) P_3_, and (**d**) P_4_ on the curve in Figure 6a.

To gain a deeper understanding of the foaming evolution process, we employed SEM to explore the distribution of powders at each stage of the temperature points ranging from P_1_ to P_4_ in Figure 6a. The corresponding observations are depicted in Figure 8. In Figure 8a, the powder composition of AFS at the P_1_ temperature point is presented. The presence of Si and Cu elements is characterized by granular morphology within the matrix, while the existence of Mg is observed in the form of oxides or as part of the Mg-Cu intermetallic compound. Progressing to the P_2_ temperature point, as shown in Figure 8b, the granular copper powder particles within the matrix completely disappear. Therefore, the turnaround observed at point M in the heating–expansion curve can be explained by the substantial heat absorption during the alloying process of copper particles with the matrix. This alignment also coincides with the vanishing of dark granularity in the synchrotron radiation field. Notably, a significant amount of silicon granularity is still evident within the matrix at this stage. Advancing to the P_3_ temperature point, slightly surpassing the E_2_′ temperature as illustrated in Figure 8c, the substantial portion of silicon powder particles remains unalloyed with the matrix. Irregular crack-like features have appeared within the matrix. Upon reaching the P_4_ temperature point, situated slightly above point N, the granular silicon powder alloy with the matrix, causing their disappearance, as depicted in Figure 8d. Pore roundness improves as the liquid-to-solid ratio of the matrix increases.

**Figure 9:**
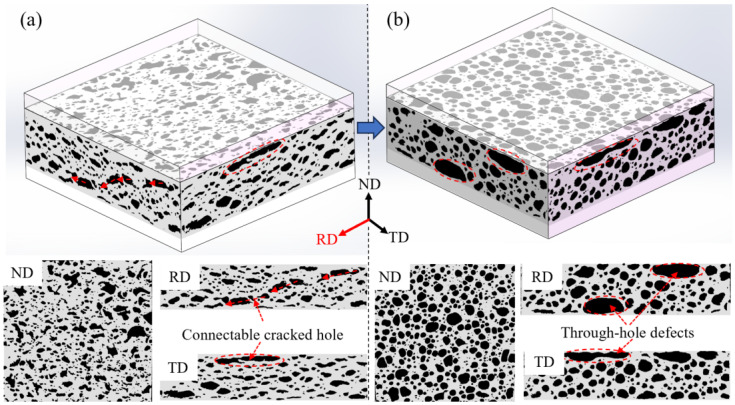
Three-dimensional structure of pore evolution process at temperature points of (**a**) P_4_ and (**b**) P_5_.

Figure 9 illustrates the three-dimensional macroscopic structural changes in pores from the P_4_ to P_5_ stages in the foaming process. In Figure 9a, the pore morphology appears irregular. The pores exhibit an oriented and flattened appearance on the cross-sections along the rolling direction (RD) and transverse direction (TD). Notably, the orientation distribution is particularly pronounced on the TD cross-section, aligned predominantly along the rolling direction of the precursor. On the RD cross-section, some pores exhibit connectable cracked features, providing pathways for internal gas to escape, thereby reducing the overall expansion rate. Furthermore, these interconnected cracks might potentially give rise to through-hole defects that are interconnected in the later stages of foaming. These defects contribute to a decline in pore uniformity, as indicated by the red wireframe in Figure 9b. Figure 9b showcases the pore structure of the precursor at the P_5_ temperature point. When compared to the AFS seen in Figure 9a, the pores’ sphericity notably increases, accompanied by a reduction in orientation distribution characteristics.

**Figure 10:**
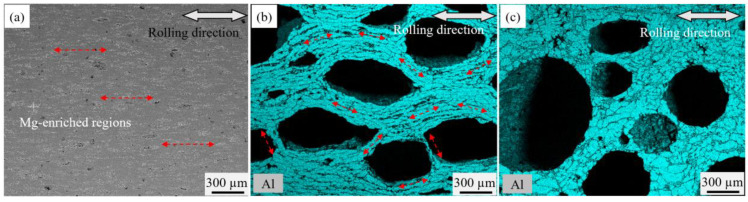
Microstructure along the rolling direction at temperatures of (**a**) P_2_, (**b**)P_4_, and (**c**) P_5_.

To unravel the pore structure evolution mechanism at the P_4_ stage, we employed SEM to observe the matrix states of different foaming stages on the transverse direction (TD) cross-section, as depicted in Figure 10. In Figure 10a, the typical structure of the precursor in its as-rolled state is presented. Due to the deformation during the rolling process, the powder distribution displays evident orientation characteristics, with elongated powder along the rolling direction (indicated by the red dashed arrows). Specifically, the component distribution within the aluminum matrix reveals a hierarchical interphase pattern perpendicular to the rolling direction. Figure 10b displays the typical structural characteristics of the aluminum matrix at the P_4_ temperature stage. At this juncture, the aluminum grains appear elongated and exhibit noticeable orientation, enveloping a state of encircling pores as indicated by the red dashed arrows. As the precursor is heated to the P_5_ temperature, the orientation of aluminum grains within the matrix completely disappears, accompanied by an increase in pore circularity, as shown in Figure 10c.

The characteristics of pore structure at different foaming stages are intricately linked to the morphology of the powder distribution. During the precursor heating process, alloying reactions tend to occur preferentially in the enriched areas for multiple elements within the powder. Notably, the contact surfaces involving various elements along the rolling direction possess the most substantial specific surface area, allowing for extensive alloying and resulting in the highest liquid-to-solid phase ratio. This is clearly illustrated in Figure 10a, where a significant amount of white Mg-enriched regions forms along the rolling direction, facilitating the initial nucleation process of cracks. Under the pressure stemming from the decomposition of titanium hydride, pores preferentially aggregate in this region, leading to the development of an oriented distribution of characteristic pore morphology. Conversely, the aluminum matrix tangent to pore boundaries impedes further pore growth and merging at this stage. These insights are evident in Figure 10b. As the temperature progresses to the P_5_ point, the orientation distribution characteristics of aluminum particles vanish due to the effective diffusion of Si, Mg, and Cu elements into the aluminum matrix. This phenomenon is demonstrated in Figure 10c, where the aluminum matrix surrounded by the second phase exhibits a granular crystal structure after cooling. Moreover, the higher liquid-to-solid ratio alleviates the matrix’s constraint on pore growth. Consequently, pores manifest a more rounded shape at this stage. Overall, these findings highlight the intricate interplay between powder distribution, alloying reactions, and temperature changes, influencing the evolving pore structure throughout the foaming process.

### 3.2. Boundary Effects and Plate Shape Modulation

**Figure 11:**
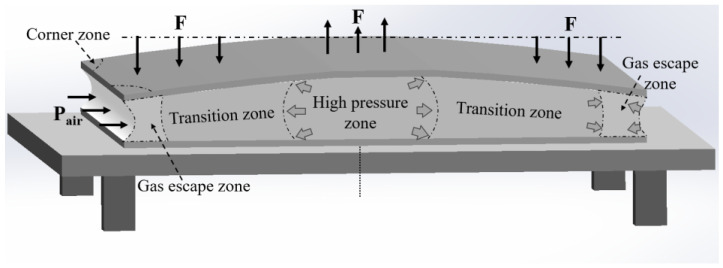
Influence of boundary effects on the characteristics of AFS plate shape.

In the preparation of large-size AFS, achieving precise control of the plate shape is a critical technical challenge that requires immediate attention. Figure 11 presents a schematic illustrating the theoretical differences in expansion ratios across various regions within the AFS. After foaming, the precursor displays diminished heights along its four sides and an increased height at its center. This outcome occurs as a result of the gases generated within the foam escaping along its edges. Specifically, the corners of the foam, which have significant contact with the external environment, experience the lowest expansion. In reality, due to the deformation resistance offered by the outer metal panel, a pressure difference occurs between regions of the foam with different expansion ratios. When this pressure difference exceeds a certain threshold, the high-pressure foam in the central region overcomes the resistance caused by the viscosity of the foam and migrates toward the low-pressure foam regions. This dynamic adjustment helps counter the effects described earlier.

For AFS with smaller panel sizes, the transitional zone between the high-pressure foam core and the low-pressure foam is short, leading to minimal resistance to flow. This allows a larger portion of foam from the central region to move toward the boundaries, effectively reducing the pressure difference. Therefore, despite the reduced expansion capacity near the larger boundary surface, the AFS still manages to maintain a flat plate shape. However, as the panel size increases, two notable factors come into play. Firstly, the precursor’s expansion capability increases when the relative boundary surface area decreases. Secondly, flow resistance increases for foam core from the high-pressure region to the low-pressure region. These combined factors result in a significant increase in the pressure difference between the boundary region and the center region of AFS. As a result, the panel becomes more susceptible to bending deformation due to increased bending moments, leading to noticeable bulging in the central region of the AFS. Additionally, the morphology of the foam core boundary is affected. Specifically, traction stresses from the outer plate layer create a low-pressure region in the boundary region, which forms a concave structure under the influence of ambient pressure. In more severe cases, external gas might penetrate the foam core at the boundary surface, leading to the formation of through-hole defects. These post-foaming AFS structural attributes are referred to as the “boundary effect”.

**Figure 12:**
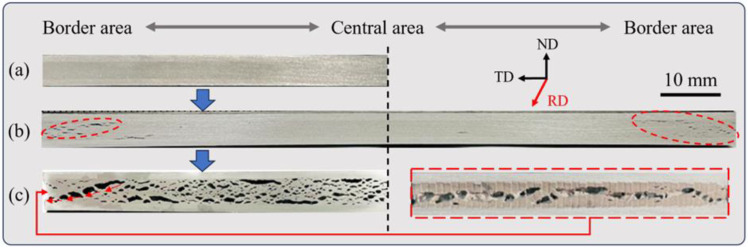
Boundary structure of different foaming stages at (**a**) P_2_, (**b**) P_3_, and (**c**) P_4_.

Especially when defects are present within the powder core layer after rolling, the challenges posed by the “boundary effect” become more pronounced. Figure 12 depicts the evolution of the AFS boundary structure across different stages, employing a titanium hydride content of less than 0.05 wt.%. In Figure 12a, serving as the reference specimens, the matrix state is depicted when the core powder is heated to point P_2_. Minimal changes are evident within the matrix at this stage. As the temperature reaches point P_3_ (as shown in Figure 12b), numerous microcracks emerge at both ends of the boundary (highlighted by red circle), while the central region of the matrix profile retains similarity to the P_2_ stage. The left edge displays more severe cracking compared to the right edge. This phenomenon further elucidates the reason for the abrupt expansion rate increase observed at point E_2_ on the temperature–expansion curve. The predilection for cracking in the boundary region can be attributed to the higher actual temperatures and weaker deformation resistance for the powder core compared to the central region. These early onsets of edge cracks amplify the contact surface between the powder in the boundary region and the external environment, thus exacerbating the detrimental effect of edge effects on the shape of the plate. Figure 12c illustrates the state when the precursor temperature reaches point P_4_, where the entire matrix exhibits numerous pore structures resembling cracks. In comparison to the central region, the boundary area displays fewer pores with a more concentrated distribution. Some of these pores interconnect, forming channels through which internal gas can escape to the external environment (indicated by the red arrows). These formations appear as structures resembling crack-like pores when observed from the exterior of the precursor, as showcased in the right red dashed box (side view of the left specimen in Figure 12c).

**Figure 13:**
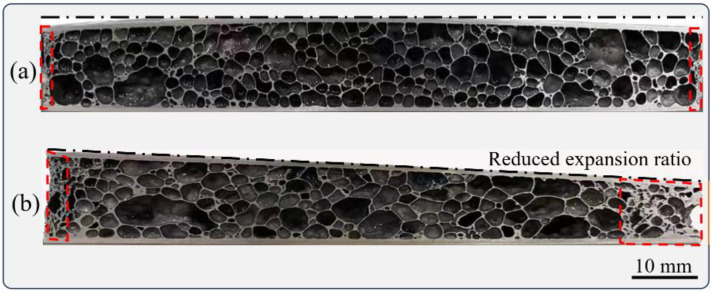
Panel defect morphology of (**a**) bending and (**b**) tilting.

The presence of the aforementioned boundary effects introduces an added layer of intricacy to the management of AFS plate shape. Figure 13 illustrates two prevalent issues concerning panel shape that arise during the AFS preparation process. In Figure 13a, the upper panel of the specimen displays suboptimal straightness, characterized by a discernible central bulge and noteworthy values for both bending and bending area. Figure 13b portrays another panel shape concern originating from boundary effects, where the upper panel inclines, and one side appears elevated compared to the other. This phenomenon can be traced back to the characteristics of the specimens shown in Figure 12b, where varying degrees of edge cracking occur during the early expansion phase of the precursor. Specifically, the differential extent of cracking at the edges on both sides leads to an uneven release of internal gases during the later stages of foaming. This imbalance ultimately results in severe tilting problems within the AFS structure.

These conclusions gain additional support when examining the boundary morphologies of the specimens. In Figure 13a, the dense aluminum layers (marked in the red dashed boxes) at both ends exhibit similar thicknesses. Conversely, Figure 13b displays a noticeable thickness discrepancy at both ends. Within powder metallurgy foaming processes, thicker, dense aluminum layers at the boundary generally correspond to a greater gas overflow. This observation further strengthens the deduction that the excessive gas spillage at the right boundary of the precursor in Figure 13b contributes to this issue. Importantly, the boundary effect curtails the expansion capacity of the matrix. 

**Figure 14:**
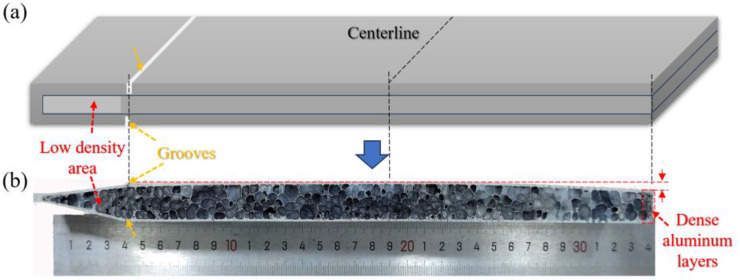
(**a**) Schematic diagrams of the precursor under different edge-sealing conditions; (**b**) cross-sectional of aforementioned AFS after foaming.

To tackle the plate shaping difficulties arising from boundary effects, a simple engineering approach involves implementing edge-sealing treatment prior to the foaming process. As depicted in Figure 14a, the precursor’s left side remains uncut, utilizing the edge-sealing technique, whereas the right side, as per traditional practice, is trimmed before foaming. In order to counteract the limitations imposed by the left boundary on matrix expansion, precise grooves are intricately carved into both the upper and lower panels within the boundary region. This deliberate design serves to guide controlled bending at this particular juncture during expansion, effectively alleviating the impact of edge-induced constraints on panel shaping. Of paramount importance, the depth of these grooves is intentionally set slightly below the panel thickness, thereby preventing the escape of gases from this area. Figure 14b depicts the cross-section of the foamed AFS. Notably, the left side showcases elevated expansion and improved flatness in comparison to the right side. Even in areas of low-density powder with poor blistering ability, the pore structure exhibits premium properties and minimizes dense aluminum layers. In contrast, on the right boundary powders, the influence of the boundary effect diminishes the expansion capacity, leading to pronounced bending in the plate shape.

## 4. Conclusions

In this study, we accomplish the engineering preparation of AFS using the powder pack rolling method and investigate the foam evolution mechanism of Al-Si-Mg-Cu alloy. Key technical issues such as cavity design and plate shape control during the preparation of large-size aluminum foam sandwich panels are analyzed and solved. The following conclusions are drawn:

(1) The precursor’s heating process is segmented into three distinct stages: rapid heating, solid–liquid transition, and stable foaming. The onset of the solid–liquid transition stage coincides with the initiation of copper powder alloying at point M, while its completion aligns with the conclusion of silicon powder alloying at point N. Within this period, the matrix expansion rate abruptly transitions from a rapid increase to a sharp decrease at point E_2_. This signifies the moment when pore nucleation occurs within the powder, accompanied by cracking at the precursor’s boundary.

(2) During the solid–liquid transition stage, the initial pore structure exhibits a crack-like pattern. Upon reaching the stable foaming stage, this structure undergoes a transformation from oriented ellipsoidal shapes to spherical formations. The elongated powder structure generated from rolling contributes to an oriented distribution of pores along the rolling direction. In the matrix composition, the Mg-enriched regions play a pivotal role in forming crackled pores at this stage. Pore roundness increases as this region alloys with matrix in the later foaming stages.

(3) During the precursor powder densification process, square tubes were used as edge seals instead of panels. This approach creates highly dense zones at the precursor boundaries, effectively halting crack propagation into the powder core. Simultaneously, it reduces the width-spreading deformation of the powder core during rolling, lowering the occurrence of low-density regions at the cavity edges and enhancing the efficient utilization of edge powder.

(4) During larger-scale AFS foaming, a noticeable boundary effect emerges, characterized by central elevation, peripheral depression, and inward concavity along the foam core’s edges. This phenomenon results from gas overflow in the boundary regions of the powder core. Initial foaming near the boundary generates edge cracks that magnify matrix–environment interaction, worsening the negative impact on plate shape. Varying degrees of edge crack severity may even lead to panel tilting. Utilizing edge sealing technology during foaming effectively prevents gas leakage, optimizing the flatness of the AFS panel post-foaming.

## Data Availability

The processed data required to reproduce these findings can not be shared at this time, as the data also form part of an ongoing study.
